# Identification of novel factors enhancing recombinant protein production in multi-copy *Komagataella phaffii* based on transcriptomic analysis of overexpression effects

**DOI:** 10.1038/s41598-017-16577-x

**Published:** 2017-11-24

**Authors:** Xiao-Wei Yu, Wei-Hong Sun, Ying-Zheng Wang, Yan Xu

**Affiliations:** The Key Laboratory of Industrial Biotechnology, Ministry of Education, School of Biotechnology, Jiangnan University, Wuxi, 214122 P.R. China

## Abstract

The methylotrophic yeast *Komagataella phaffii* (*Pichia pastoris*) has been developed into a highly successful system for heterologous protein expression in both academia and industry. However, overexpression of recombinant protein often leads to severe burden on the physiology of *K. phaffii* and triggers cellular stress. To elucidate the global effect of protein overexpression, we set out to analyze the differential transcriptome of recombinant strains with 12 copies and a single copy of phospholipase A_2_ gene (*PLA*_2_) from *Streptomyces violaceoruber*. Through GO, KEGG and heat map analysis of significantly differentially expressed genes, the results indicated that the 12-copy strain suffered heavy cellular stress. The genes involved in protein processing and stress response were significantly upregulated due to the burden of protein folding and secretion, while the genes in ribosome and DNA replication were significantly downregulated possibly contributing to the reduced cell growth rate under protein overexpression stress. Three most upregulated heat shock response genes (*CPR6*, *FES1*, and *STI1*) were co-overexpressed in *K. phaffii* and proved their positive effect on the secretion of reporter enzymes (PLA_2_ and prolyl endopeptidase) by increasing the production up to 1.41-fold, providing novel helper factors for rational engineering of *K. phaffii*.

## Introduction

Over the last few decades, *Komagataella phaffii* (formerly known as *Pichia pastoris*) expression system has been used successfully for production of various recombinant heterologous proteins^1,2^. Many factors can potentially affect heterologous protein production in *K. phaffii*, such as promoter, gene dosage, gene sequence, and post-translational modification of proteins^3^. In this context, the alcohol oxidase (*AOX1*) promoter is the most widely used promoter, which is tightly and strongly regulated by methanol^4^. Gene dosage is a critical strategy for maximizing expression in *K. phaffii* ^2,5^. The expression level of *Rhizopus chinensis lipase* was improved by 6.2-fold by increase gene copy number from one to five in *K. phaffii* ^6^. The mRNA and protein expression levels of Proteinase K gene increased as gene dosage increased in *K. phaffii* ^7^. However, increased recombinant gene dosage beyond a certain threshold proves to be a severe burden on the protein folding and secretory machine, resulting in lower productivity of target protein^6,8^.

Overexpression of recombinant protein often leads to a misfolded product and triggers cellular stress. The cellular response to stress is represented at the molecular level by inducing the synthesis of heat shock proteins (HSPs), including two classes, molecular chaperones and proteases. Molecular chaperones functioning in protein folding, translocation, and refolding of intermediates are directed toward the capture of folding intermediates to prevent misfolding and aggregation and to facilitate refolding or degradation^9^. Proteases ensure that damaged and short-lived proteins are degraded efficiently^9^. The protein folding in the endoplasmic reticulum (ER) often presents a bottleneck in the eukaryotic secretion pathway, leading to the unfolded protein response (UPR) and the ER-Associated Degradation (ERAD) pathway^10^.

Coexpression of helper genes or molecular chaperones has been used to improve the heterologous expression in *K*. *phaffii* by overcoming the burden of protein folding and secretion. One example is Kar2, which binds many proteins during translocation through the translocon pore into the lumen of ER, Ero1 and Pdi, catalyzing the oxidation, reduction, and isomerization of disulfide bonds, ssa4, targeting nascent proteins to ER, and the unfolded protein response transcription factor Hac1^4,11–18^. The effects of coexpression on the target protein can vary depending on the molecular chaperones. Coexpression of Pdi did not show any beneficial effect on horseradish peroxidase (HRP) production, indicating that disulfide bridge formation was not limiting HRP secretion. However, when coexpressed with Hac1 an increased HRP titer was observed^15^. Besides the often used transcription factor Hac1, Ruth *et al*.^16^ demonstrated that a novel *K. phaffii* transcription factor Aft1 can be used to improve recombinant protein secretion, which enhanced the secretion of a carboxylesterase by 2.5-fold. After screening ten helper genes involved in protein folding and stress response, the yield of a glucose oxidase was improved by 5.11-fold in *K. phaffii* when coexpressed with three chaperones, Sec. 53, Cne1 and Gcn4^13^. These studies demonstrate that genetic engineering of *K. phaffii* through helper factors has been served as valuable tools for maximizing protein production.

Novel helper factors have been discovered by “omics” for improvement of recombinant protein production^14,19–24^. Six novel secretion helpers (Bmh2, Bfr2, Ssa4, Sse1, Cup5 and Kin2) were selected by comparison the DNA microarray data between a recombinant protein producing *K. phaffii* strain and a nonproducing strain, and coexpressed to test the potential to improve the heterologous protein expression^19^. Several helper factors, involved in oxidative stress response, methanol metabolism, glycolytic pathway, and protein folding, were identified in *K. phaffii* based on transcriptomic and proteomic analysis under simulated microgravity^20,21^. The physiological hypoxia adaptation of the recombinant *K. phaffii* strain expressing a Fab antibody fragment was analyzed by transcriptomic, proteomic and metabolic fluxes^25^. Based on this research eight potential target genes were tested for strain improvement, among which, Wsc4, involved in trafficking through the ER, showed a beneficial effect on the product yield with a 1.2-fold increase^22^. Vogl *et al*.^14^ studied the transcriptional response to the production of three ER-resident membrane proteins using DNA microarrays. And then, the expression levels of the membrane proteins were increased up to 2.1-fold by coexpression with Hac1. Three secretion enhancing factors were identified out of a cDNA library by cell surface display and fluorescent-activated cell sorting (FACS), increasing the relative expression level of the model protein up to 45%^23^. Through genome scale metabolic modelling of *K. phaffii*, the helper genes enhancing recombinant protein production were identified by overexpressing or knocking out the targets involved in primary metabolism^24^.

Foreign protein production in *K. phaffii* has a great influence on cell physiology. Functional helper factors are expected to be significantly induced in strains especially under the stress of overexpression. However, the identification of potential helper factors based on the comparing the transcriptional profile during strong overexpression versus low production level of a recombinant protein in *K. phaffii* has not yet been explored. In addition, high titer (>1 g/L) is required for the production of recombinant protein in industrial scale^26^. Thus, a more detailed understanding of the physiology of *K. phaffii* by comparison of global transcription of high- and low- titer producing strain is urgently needed, through which novel helper factors could be discovered. In this study, for the first time, we performed global transcriptomic analyses of the recombinant *K. phaffii* strains with 12 copies and a single copy of phospholipase A_2_ gene (*PLA*_2_) from *Streptomyces violaceoruber*, which produced two levels of PLA_2_ in titer over 1g/L, and then evaluated the effect of the potential helper genes on recombinant protein secretion by gene coexpression.

## Materials and Methods

### Strain construction

Recombinant *K. phaffii* strains integrated with different copies of *PLA*_2_ gene were constructed as described in our previous paper^27^. *K. phaffii* strains GS115/pPIC9K-PLA2-12 with 12-copy gene and GS115/pPIC9K-PLA2-1 with 1-copy gene were used for RNA-Seq. The *PLA*_2_ gene was integrated into the genome of *K. phaffii* GS115 under the control of *AOX1* promoter. The secretion signal peptide α-factor of pPIC9K was used as the secretion signal. The *PLA*_2_ gene copy number was determined according to Sha *et al*.^6^. The strain GS115/pPIC9K, which integrated with the vector pPIC9K without heterologous genes, was used as a negative control. The strains and samples used for RNA-Seq are summarized in Table 1. Two biological replicates of each sample were analyzed.

**Table 1:** Sample information for RNA-Seq^&^.

Sample name*	Strain/plasmid	*PLA*_*2*_ gene copy number	PLA_2_ protein concentration (g L^−1^)^#^	PLA_2_ Enzyme activity (U mL^−1^)	Relative *PLA*_2_ transcription level	µ (h^−1^)	q_s_ (g_methanol_ g_DCW_^−1^ h^−1^)	q_p_ (U g_DCW_^−1^ h^−1^)
MC	GS115/pPIC9K-PLA2-12	12	5.7 ± 0.4	720 ± 34	4.5 ± 0.3	0.015 ± 0.003	0.17 ± 0.02	0.11 ± 0.01
SC	GS115/pPIC9K-PLA2-1	1	1.1 ± 0.3	115 ± 20	1.0 ± 0.2	0.031 ± 0.005	0.14 ± 0.02	0.04 ± 0.01
BK	GS115/pPIC9K	0	—	—	—	0.029 ± 0.004	0.12 ± 0.02	—

*Sampling at 48 h after methanol induction in fed-batch fermentation.

^&^Data are the mean and standard deviation of three biological replicates. Two biological replicates of each sample were used for RNA-Seq. µ: specific growth rate; q_s_: specific substrate (methanol) consumption rate; q_p_: specific product formation rate; DCW: dry cell weight; µ, q_s_ and q_p_ were calculated during the methanol induction time from 0 to 48 h.

^#^The total protein concentration in the supernatant was determined by *the* Bradford method, and then the *PLA*_2_ concentration was calculated according to the percentage of *PLA*_2_ on the SDS-PAGE by the Quantity One software.

The strain GS115/pPIC9K-MLMH expressing *Aspergillus oryzae* prolyl endopeptidase fused with maltose binding protein (MLMH) with one gene copy number was constructed in our lab^28^. The expression plasmids containing helper genes (*CPR6*, *FES1*, *STI1*, and *SSA1*) were constructed by inserting the coding sequence into the vector pPICZA (Invitrogen, USA) for intracellular expression under the control of *AOX1* promoter. *K. phaffii* strains GS115/pPIC9K-PLA2-12, GS115/pPIC9K-PLA2-1 and GS115/pPIC9K-MLMH were transformed with the linearized expression plasmids using electroporation (Multi-Copy *Pichia* Expression Kit, Invitrogen), and the strains transformed with empty vector pPICZA were used as negative control. The gene copy number of the helper gene was determined^6^ and the strains containing only one copy of helper gene were selected for heterologous protein production.

### Gene copy number determination

The gene copy number was determined by real-time quantitative PCR (qPCR) described by Sa *et al*.^6^. The designed primers were annealed to the complementary regions of the *AOX1* promoter sequence. The parent strain GS115 contains only one *AOX1* promoter. Thus, the copy number of *P*_*AOX1*_ minus one equals the copy number of the gene of reporter enzyme controlled under the *AOX1* promoter. qPCR data were normalized using GAPDH gene as the endogenous control (reference gene). All qPCR reactions were run in triplicate on MJ chromo4 (MJ, America) using the following program: 98 °C 2 min, 40 cycles of 98 °C for 5 s, and 50 °C for 5 s. The copy number of the helper gene was also determined using this method.

### Shake flask cultivation

Shake flask cultivation was performed as described by Liu *et al*.^27^. Briefly, an individual colony was inoculated in BMGY medium (10 g/L yeast extract, 20 g/L peptone, 100 mM potassium phosphate pH6, 13.4 g/L yeast nitrogen base with ammonium sulfate without amino acids, 0.4 mg/L biotin, 1% (v/v) glycerol) shaken at 30 °C and 200 rpm. When cultures reached an OD_600_ of about 6, the cells were centrifuged and resuspended in BMMY medium (10 g/L yeast extract, 20 g/L peptone, 100 mM potassium phosphate pH6, 13.4 g/L yeast nitrogen base with ammonium sulfate without amino acids, 0.4 mg/L biotin, 0.5% (v/v) methanol) to an OD_600_ of 1.0, shaken at 28 °C and 250 rpm for 120 h. The cultures were supplemented with methanol (10 g/L) to induce the heterologous protein expression every 24 h. All reactions were performed in triplicate.

### Fed-batch fermentation

Cultivation were performed in a 7-L bioreactor (New Brunswick, BioFlo 110, Edison, N.J., USA) according to Yu *et al*.^29^ with some modification. The fermentation process includes three phases: glycerol batch phase, glycerol fed-batch phase and methanol induction phase. Basal Salts Medium (BSM) was used for fed-batch fermentation, composed of 40 g/L glycerol, 22.7 g/L H_3_PO_4_, 0.93 g/L CaSO_4_, 18.2 g/L K_2_SO_4_, 14.9 g/L MgSO_4_ ·7H_2_O, 4.13 g/L KOH, 7.0 g/L K_2_HPO_4_ and 4 mL/L trace solution (6 g/L CuSO_4_·5H_2_O, 0.08 g/L NaI, 3.0 g/L MnSO_4_ · H_2_O, 0.2 g/L Na_2_MoO_4_ ·2H_2_O, 0.02 g/L H_3_BO_3_, 0.5 g/L CoCl_2_, 20 g/L ZnCl_2_, 65 g/L FeSO_4_·7H_2_O, 0.2 g/L biotin, and concentrated sulfuric acid, 0.5% (v/v)). The pH of the medium was adjusted and controlled at 5.5 with the addition of 28% (v/v) ammonia solution during the whole cultivation period. In the methanol induction phase, the methanol concentration was controlled at 0.1 ± 0.02% (v/v) by an on-line methanol analyzer (FC2002, Shanghai Super-xinxi, China). The dissolved oxygen concentration was kept above 20% by controlling the stirred speed between 600 and 1200 rpm at a constant airflow of 150 L/h. All reactions were performed in triplicate.

### Determination of PLA_2_ activity

The activity of PLA_2_ was determined as described by Liu *et al*.^27^ using acid-base titration methods. One unit of PLA_2_ activity corresponds to the amount of enzyme which releases 1 µmol of free fatty acids per minute using soybean lecithin as substrate at 50 °C and pH 6.0.

### Determination of protein concentration

Protein concentration was determined according to the method of Bradford with bovine serum albumin as a standard^30^. Semi-quantitative determination of the recombinant protein concentration in the supernatant was analyzed according to the percentage of the recombinant protein on the SDS-PAGE, quantified by the Quantity One software (Bio-Rad).

### RNA sequencing and data analysis

Total RNA was isolated by using TRIzol reagent (Invitrogen, Carlsbad, CA, USA), according to the manufacturer’s instructions. A total amount of 3 μg RNA per sample was used as input material for the RNA sample preparations. Firstly, ribosomal RNA was removed by Epicentre Ribo-zero™ rRNA Removal Kit (Epicentre, USA), and rRNA free residue was cleaned up by ethanol precipitation. Subsequently, sequencing libraries were generated by using the rRNA-depleted RNA by NEBNext® Ultra™ Directional RNA Library Prep Kit for Illumina® (NEB, USA) following the manufacturer’s recommendations. Strand-specific sequencing was performed to prepare RNA library. The libraries were sequenced on an Illumina Hiseq. 4000 and 150 bp paired-end reads were generated. Two biological replicates of each sample were analyzed. Clean data (clean reads) were obtained by removing reads containing adapter and reads of low quality from raw data. Reference genome and gene model annotation files were downloaded from genome website (https://www.ncbi.nlm.nih.gov/genome/genomes/22977)^31–33^. Index of the reference genome was built using Bowtie v2.0.6^34^ and paired-end clean reads were aligned to the reference genome using TopHat v2.0.9^35^. The mapped reads of each sample were assembled by Cufflinks v2.1.1^35^ in a reference-based approach. The gene expression level was normalized by the number of fragments per kilo-base of exon per million fragments mapped (FPKM)^36^. For statistical analysis, *p-*value < 0.05 was set as the threshold for significantly differential expression calculated by Cuffdiff v2.1.1^36^.

### GO, KEGG and heat map analysis

Gene Ontology (GO) enrichment analysis of differentially expressed genes were implemented by the GOseq R package^37^, in which gene length bias was corrected. A GO category was considered significantly enriched when its FDR-corrected *p*-value was lower than 0.05. The KOBAS software was used to test the statistical enrichment of differentially expressed genes in KEGG pathways (http://www.genome.jp/kegg/)^38^. Heat maps were created with RStudio (https://www.rstudio.com/) using differentially expressed genes.

### RT-qPCR (reverse transcription-qPCR)

Total RNAs were used for the first strand cDNA synthesis according to manufacturer’s instruction (PrimeScript™ II 1st Strand cDNA Synthesis Kit). Next, the cDNA library was subjected to quantification of the transcription level of target mRNA with β-Actin as an endogenous control by using a standard SYBR Green PCR kit (Takara) on the StepOnePlus Real-time PCR detection system (Applied Biosystem). The quantitative PCR was performed under the following conditions: 95 °C 2 min, 40 cycles of 95 °C for 5 s, and 60 °C for 30 s. 2^−ΔΔCT^ method was used to quantify mRNAs expression level. All reactions were performed in triplicate for each sample.

### Data Availability

The datasets generated during and/or analysed during the current study are available in the DDBJ (DNA Data Bank of Japan) with the accession number DRA005611.

## Results and Discussion

### Effect of gene dosage on the production of PLA_2_ by *K. phaffii*

In order to select suitable multi-copy strains for RNA-seq analysis we evaluated the effect of gene dosage on the production of PLA_2_ by *K. phaffii*. Clones with abnormal morphology were discarded, which usually indicates off-target integration^39^. In consideration of clonal variation^40^, eight clones for each multi-copy strain were cultured in shake flasks. In order to minimize the disturbance of clonal variation, two clones showing the highest and the lowest enzyme activity in the supernatant were discarded. As shown in Fig. S1, the PLA_2_ activity at 84 h after methanol induction is positively correlated with gene copy number. The PLA_2_ activity in the 12-copy strain is relative higher than those of other multi-copy strains, except for the 16-copy strain. Although the enzyme activity in the 16-copy strain is the highest, the experimental data is much away from the theoretical value calculated from the equation fitting between gene copy number and enzyme activity. Thus, recombinant strains carrying 1 (SC) and 12 (MC) copies of *PLA*_2_ gene were selected for RNA-seq analysis. The strains (SC and MC) were grown in fed-batch cultures involving initial growth on glycerol to increase biomass, followed by a switch to methanol fed-batch growth, with methanol concentration measured with a dedicated probe and controlled at approximately 0.1% (v/v). Upon addition of methanol, the expression of *PLA*_2_ gene was induced under the *AOX1* promoter and methanol was served both as inducer and carbon source. Fed-batch cultivation instead of growth in chemostat culture was employed in order to capture the regulation of a typical industrial production process in response to overexpression of recombinant protein expression although the data obtained is inherently more noisy. All samples were collected at 48 h after methanol induction for RNA-seq analysis. GS115 transformed with empty vector pPIC9K was used as the negative control (BK).

**Figure 1:**
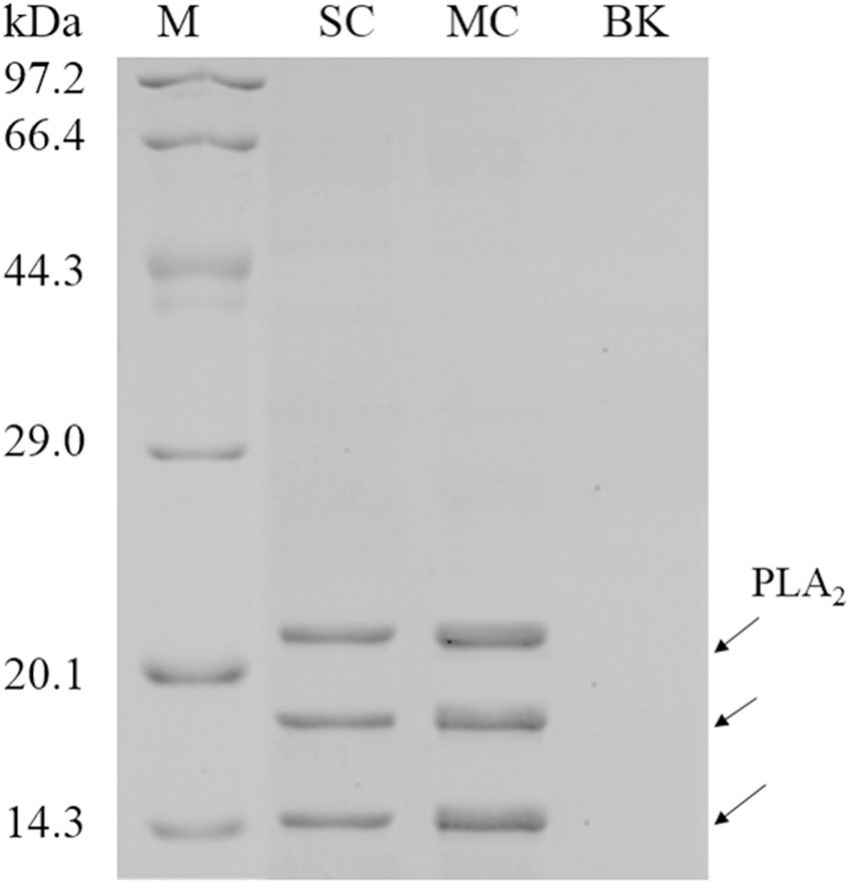
SDS-PAGE of total protein in the supernatant at 48 h after methanol induction in fed-batch fermentation of GS115/pPIC9K-PLA2-12 (MC), GS115/pPIC9K-PLA2-1 (SC) and GS115/pPIC9K (BK, as negative control). Arrows indicates the recombinant PLA_2_. The sample of MC was diluted by three times before loading.

As shown in Table 1 and Fig. S2, after 48 h of induction the PLA_2_ protein concentration and enzyme activity in the supernatant and the transcription level of *PLA*_2_ in MC were 5.2-, 6.3- and 4.5-fold higher than those in SC, respectively. On the SDS-PAGE loaded with culture supernatant (Fig. 1), the protein concentration of PLA_2_ in MC (diluted by three times) was about 2-fold higher than that in SC quantified by the Quantity One software and the three bands indicated by arrows were proved to be PLA_2_ with different degrees of N-glycosylation by peptide mass fingerprinting analysis^27^. Both the specific methanol consumption rate (q_s_) and the specific product formation rate (q_p_) in MC were higher than those in SC (Table 1). These results indicated that the production of PLA_2_ in the 12-copy strain was much higher than that in the 1-copy strain. However, after normalizing the data by copy number we found that the normalized PLA_2_ protein concentration, enzyme activity and the transcription level in MC were 2.3-, 1.9- and 2.7-fold lower than those in SC, implying an inefficient *PLA*_2_ transcription. In addition, a decrease in specific growth rate (µ) in MC was observed. These results suggested that the strain with high copy gene number of *PLA*_2_ suffered a great impact in physiology due to the stress of overexpression of PLA_2_. As indicated in the review on the effect of gene copy number on protein titer in *K. phaffii* ^5^, when secretion saturation occurs, a bottleneck occurs due to increased traffic through the secretory pathway, whereby increasing gene copy number does not always equate to higher titer. Cámara *et al*.^8^ also observed a reduced recombinant protein transcription and a slower cell growth in high-copy strains.

### Global transcriptional profiling of multi-copy recombinant *K. phaffii*

**Figure 2:**
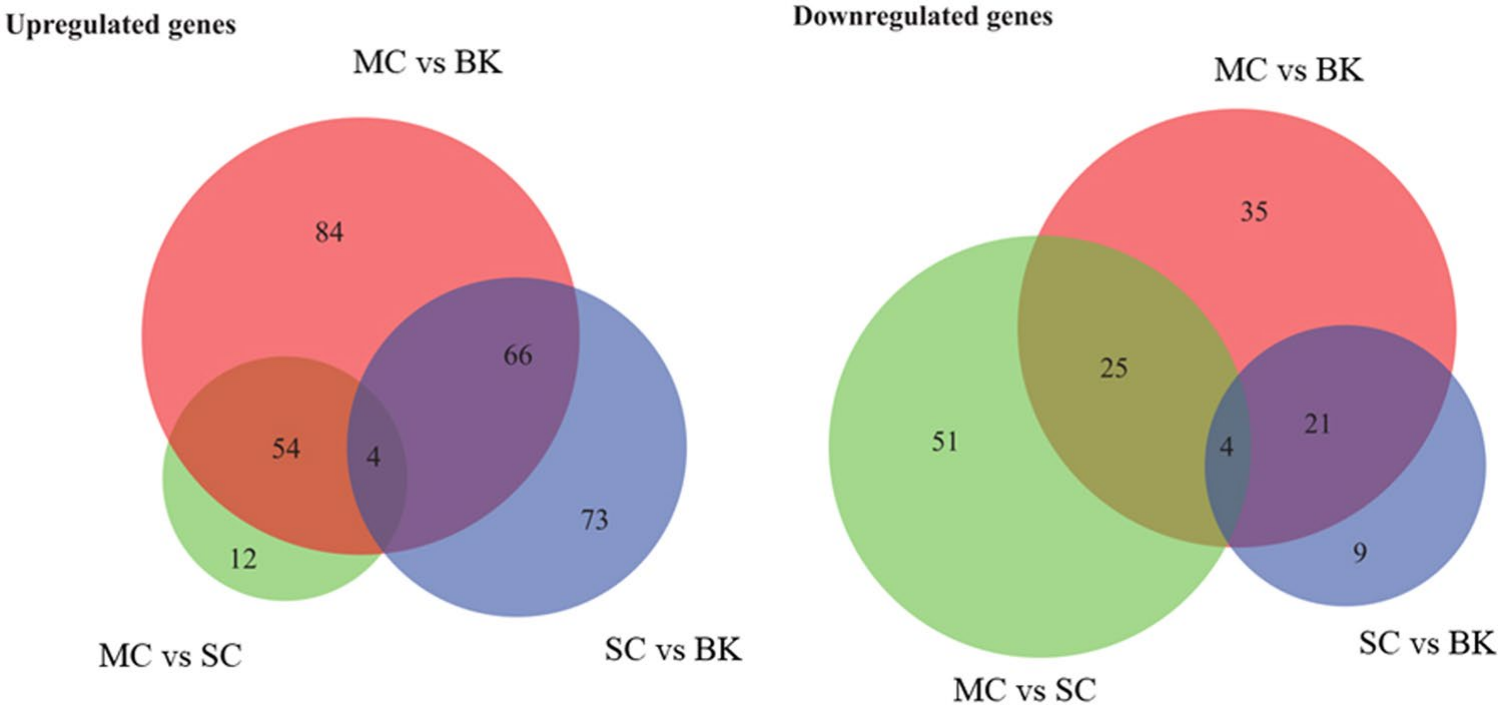
Venn diagram. Venn diagram illustrates the relationship of up- and down-regulated annotated genes (*p* < 0.05) in MC, SC and BK at 48 h after methanol induction in fed-batch fermentation.

In total 4714 transcripts were identified by RNA-seq, covering 93% protein-encoding genes annotated in the 9.46Mbp genomic sequence of *K. phaffii* GS115 strain^31–33^. The raw sequencing data were deposited in DDBJ (DNA Data Bank of Japan) with the accession number DRA005611. The RNA-seq data were validated by RT-qPCR for a subset of 7 significantly upregulated genes and 3 downregulated genes in MC vs SC. The results are shown in Fig. S3 and indicates a good correlation (R^2^ = 0.93) between RNA-seq and RT-qPCR data. Expression levels of 438 genes changed significantly (*p* < 0.05) in comparison of three data sets (MC vs BK, SC vs BK, MC vs SC). Among these differentially regulated genes, 293 genes were significantly upregulated, while 145 genes were significantly downregulated. As illustrated in the Venn diagrams (Fig. 2), the largest number of regulated genes appeared to be in the upregulated genes in the MC vs BK comparison. Furthermore, the higher the *PLA*_2_ gene copy number the more the differentially regulated genes when compared to the non-expressing control strain (BK). The upregulated and downregulated genes in MC vs BK were 4.4% and 3.0% in total transcripts, respectively, which were 2.6% and 2.3% higher than those in SC vs BK, respectively. Analysis of the overlap in the regulated genes between MC vs BK and SC vs BK indicated that 95 genes were differentially expressed. To further evaluate the impact of *PLA*_2_ dosage on the expression pattern, the 12-copy strain and the 1-copy strain were compared. In this case, 80 genes were downregulated and 70 genes were upregulated. These first results of the comparative analysis already pointed to major differences in gene expression in response to variable foreign gene production in *K. phaffii*.

**Figure 3:**
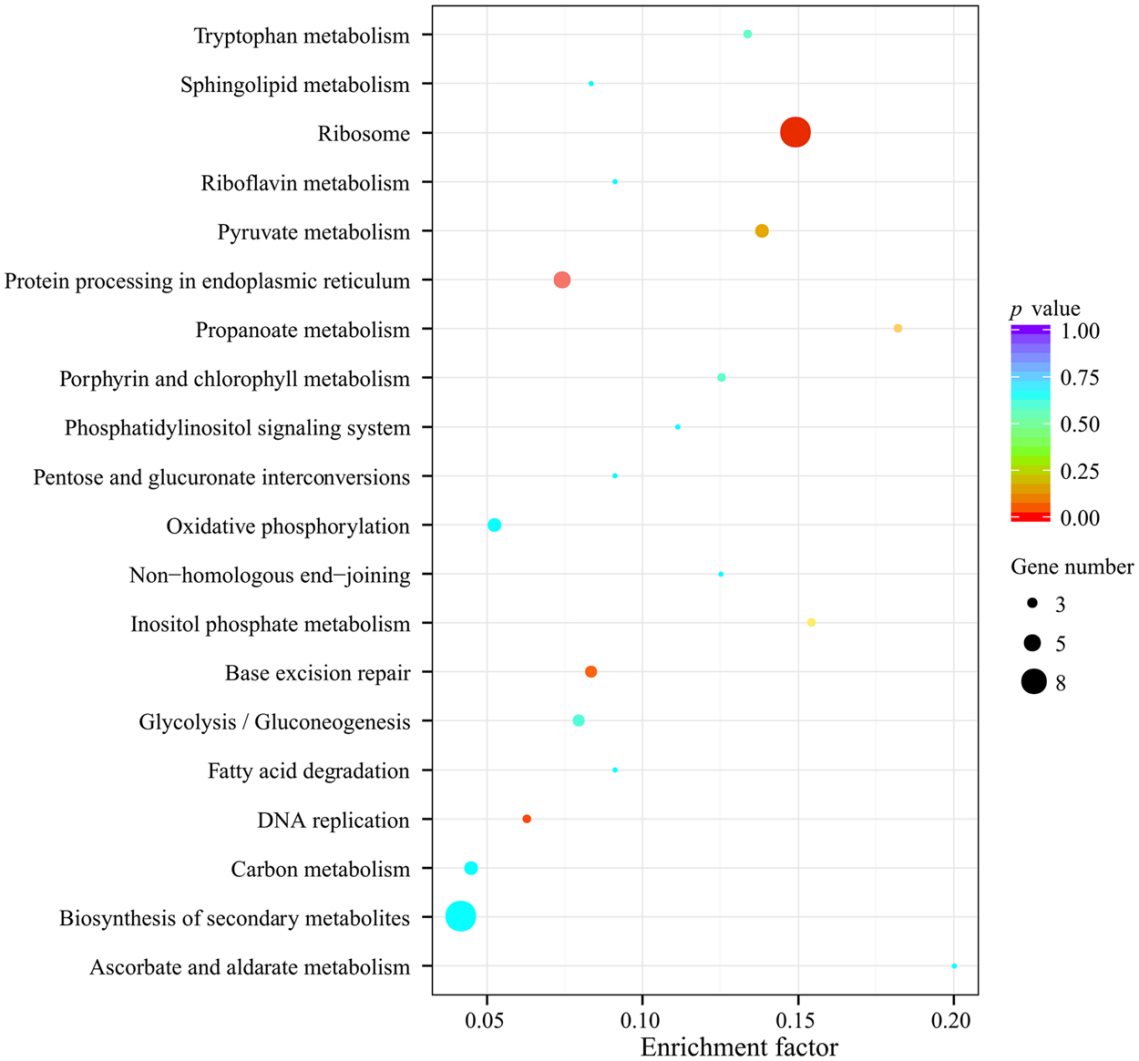
Scatterplot of enriched KEGG pathways for the differentially expressed genes (*p* < 0.05) between MC and SC. Gene number refers to the number of differentially expressed genes annotated in this KEGG pathway term. The enrichment factor is the ratio of differentially expressed gene numbers annotated in this pathway term to all gene numbers annotated in this pathway term. The transcription levels of genes by RNA-seq were determined at 48 h after methanol induction in fed-batch fermentation of MC and SC.

**Figure 4:**
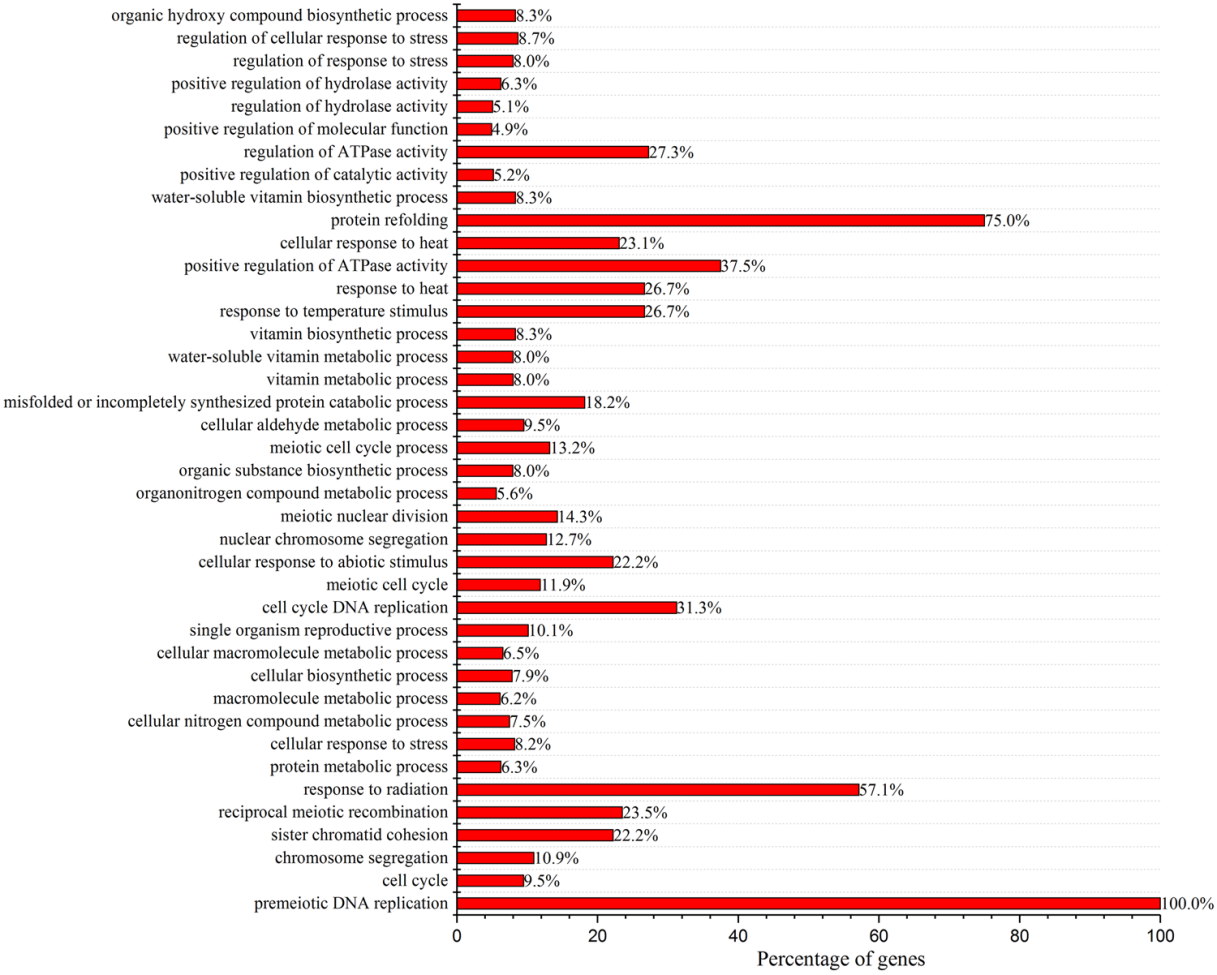
GO biological process analysis for the differentially expressed genes (*p* < 0.05) between MC and SC. X-axis indicates the percentage of differentially expressed gene numbers annotated in this GO term to all gene numbers annotated in this GO term. The transcription levels of genes by RNA-seq were determined at 48 h after methanol induction in fed-batch fermentation of MC and SC.

The KEGG metabolic pathway analysis (Fig. 3) of the 150 differentially expressed genes (MC vs SC) indicated that the gene functions were mainly enriched (*p* < 0.05) in ribosome, protein processing in ER, base excision repair, and DNA replication. In addition, GO biological process analysis (Fig. 4) revealed that the genes are especially involved in premeiotic DNA replication, protein refolding, stress response (response to radiation, response to heat, response to temperature stimulus) and regulation of ATPase activity. These analyses indicated that *K. phaffii* drastically altered the manner of gene expression in response to different recombinant protein production. Taking into consideration both GO and KEGG analysis, the genes involved in protein processing, stress response (heat shock response), ribosome and DNA replication were further analyzed using heat maps.

### Heat map analysis of genes in ribosome and DNA replication

**Figure 5:**
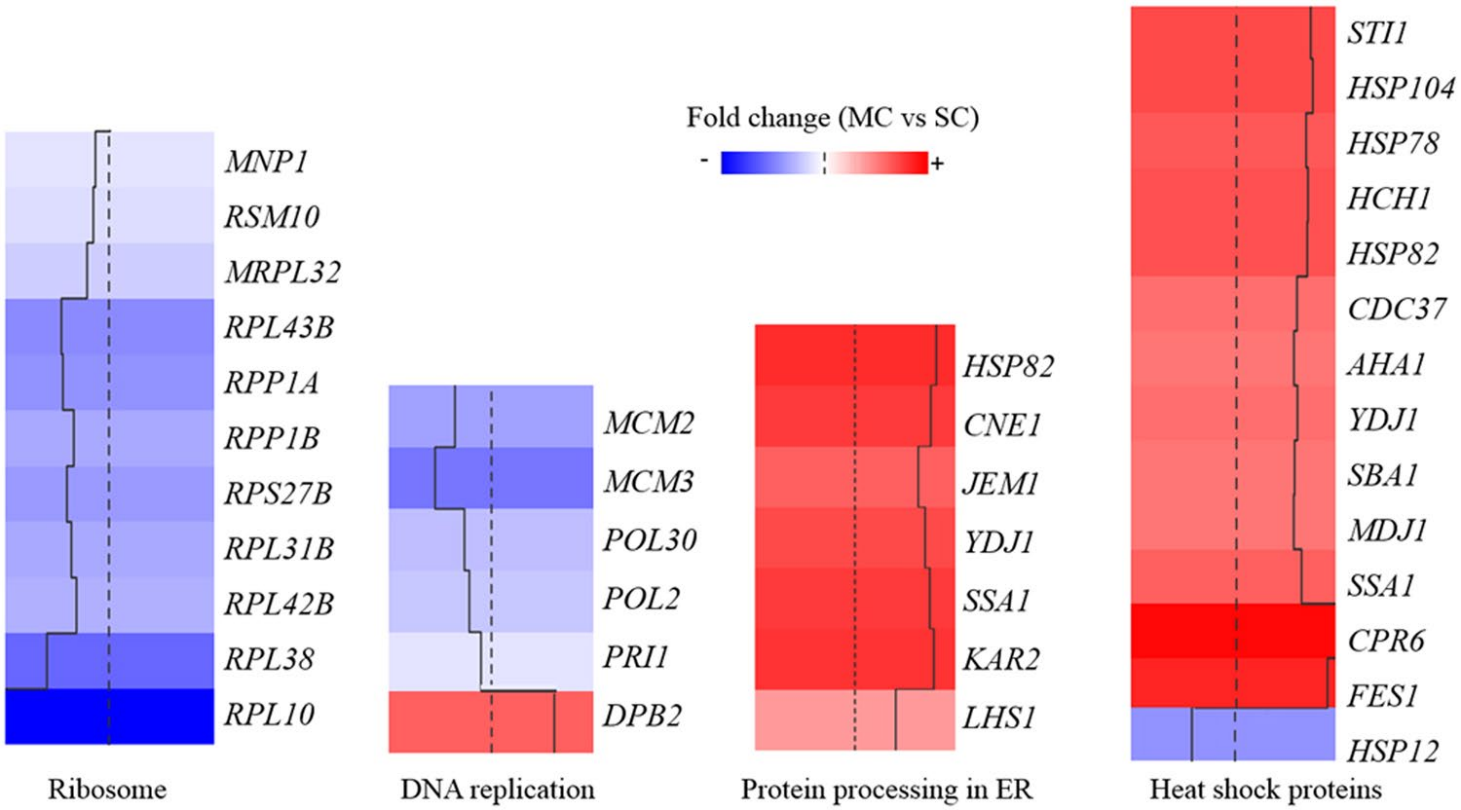
Heat map of the differentially expressed genes (*p* < 0.05) in MC vs SC involved in ribosome, DNA replication, protein processing and heat shock response. The transcription levels of genes by RNA-seq were determined at 48 h after methanol induction in fed-batch fermentation of MC and SC. The gene names are according to the corresponding sequence in *S. cerevisiae*. The gene description and the gene locus tag in *K. phaffii* are listed in supplementary Table S2.

The expression levels of almost all significantly differentially expressed genes involved in ribosome and DNA replication in MC were lower than those in SC (Fig. 5). Edwards-Jones *et al*.^41^ observed a reduction in growth rate in the 3-copy *K. phaffii* strain expressing human trypsinogen-1 compared to the 1-copy strain and a non-producing control, and analysis of the microarray information indicated that the transcript levels of ribosome-related genes and DNA replication genes were positively correlated with the cell growth rate, which was also noticed in *Saccharomyces cerevisiae* ^42^. By proteomics the ribosomal proteins were found to increase in proportion under faster cell growth rate^43,44^. In our study, the specific growth rate of high copy strain (MC) was about two-fold slower than that of low copy strain (SC). The downregulated expression of genes involved in DNA replication and ribosome in MC vs SC might be partially in response to the reduction in growth rate. Approx. 90% of the total cell energy in exponentially growing yeast was estimated to be used in ribosome production^45^. DNA replication is the critical process before cell division can take place. Thus, it is reasonable that the synthesis of ribosomal proteins and DNA replication enzymes were reduced along with the decrease of cell growth rate. However, when there was no significant variation in the growth rates between the recombinant strains and the non-producing strain, upon heterologous protein expression the transcription of ribosomal proteins was upregulated, indicating an increased translation demand for higher levels of recombinant protein production^40^. Therefore, ribosomal biogenesis is tightly controlled and regulated based on cell growth rate and protein production.

### Heat map analysis of genes in protein processing and heat shock response

As shown in Fig. 5, when analysis of the significantly differentially expressed genes in protein processing in ER and heat shock response, the expression levels of all genes except for one in MC were higher than those in SC. The upregulated genes were predicted to be in response to the stress of protein overexpression since the secreted foreign protein PLA_2_ in MC was much higher than that in SC. The four most upregulated genes in the category of protein processing in ER and HSPs were listed in Table S1. In protein processing in the ER, the most upregulated gene was *HSP8*2 with an increase of 6.26-fold, followed by *KAR*2 (known as UPR marker)*, CNE1* and *SSA1*. In HSPs, the transcription levels of *CPR6*, *FES1*, *HSP104* and *STI1* were increased by 13.98-, 10.56-, 7.22- and 6.84-fold, respectively. These results were in agreement with several previously reported cases where cellular stress response was activated during the expression of recombinant proteins in yeast^41,46,47^. In eukaryotic cells, ER is the first organelle that secretory proteins pass through, and is known as a protein-folding compartment. Many kinds of enzymes and molecular chaperones reside in the ER to assist protein folding and assembly^48^. In response to protein overexpression, the synthesis of ER resident chaperones and enzymes is induced to facilitate protein folding and/or to prevent protein aggregation^49^. Heat-shock proteins are upregulated under stress conditions in which the concentrations of aggregation-prone folding intermediates increase^50^. Nevertheless, Cámara *et al*.^8^ demonstrated that ER stress was not globally activated in the multi-copy strains compared to the non-producing control growing under steady-state conditions since the regulation of UPR, secretion and folding genes was not intense, with a maximum fold change of 2. This is in contrast to the observations in our study, which may be as a result of the different cultivation conditions. Although both studies explored the transcriptomics of multi-copy strains producing recombinant protein, we used a fed-batch process rather than the steady-state condition of Cámara *et al*.^8^. Fed-batch fermentation is commonly used in industrial processes for recombinant protein production, usually having advantage of higher product titer over steady-state condition^51^. Under fed-batch fermentation, cells often suffer from more environmental stresses from high cell density cultivation and nutrient limitation compared to the steady-state condition^51,52^, in turn triggering heat shock response to recombinant protein production in *K. phaffii*. However, it is also possible that production intensity and different recombinant targets have impacts on the cellular stress response.

Functioning as molecular chaperones, HSPs have great overlap with protein processing proteins in ER. All eight upregulated genes were molecular chaperones, among which seven except for Calnexin (Cne) were HSPs. Hsp82 belongs to Hsp90 family, while Ssa1 and Kar2 belong to Hsp70 family. Cpr6, Fes1, Hsp104 and Sti1 interact with the Hsp90/Hsp70 chaperone machine^53^. The Hsp90/Hsp70 chaperone machine is an essential regulator of cell growth and division. It is required for activation of select client proteins, chiefly protein kinases and transcription activators and thus plays a major role in regulating intracellular signaling and gene expression^54^. Both co-chaperones Cpr6 and Sti1 containing tetratricopeptide repeat (TPR) domains function as regulators of the ATPase activity of Hsp90^55^. The nucleotide exchange factor Fes1 interacts *in vivo* preferentially with the Ssa family of cytosolic Hsp70, which plays critical roles in protein homeostasis^56^. Hsp104 plays a central role in the clearance of aggregates after heat shock and the propagation of yeast prions^57^. Cne is an integral membrane ER chaperone involved in quality control of glycoproteins, routing proteins to Golgi, ERAD, or keeping them in the folding process^58^. Focusing on upregulated genes, which have potential functions in protein folding and stress regulation, three most upregulated genes (*CPR6*, *FES1*, and *STI1*) were selected for further analysis of their effects on the recombinant protein production by co-overexpression methods.

### Effect of coexpression of helper genes on heterologous protein production by *K. phaffii*

**Figure 6:**
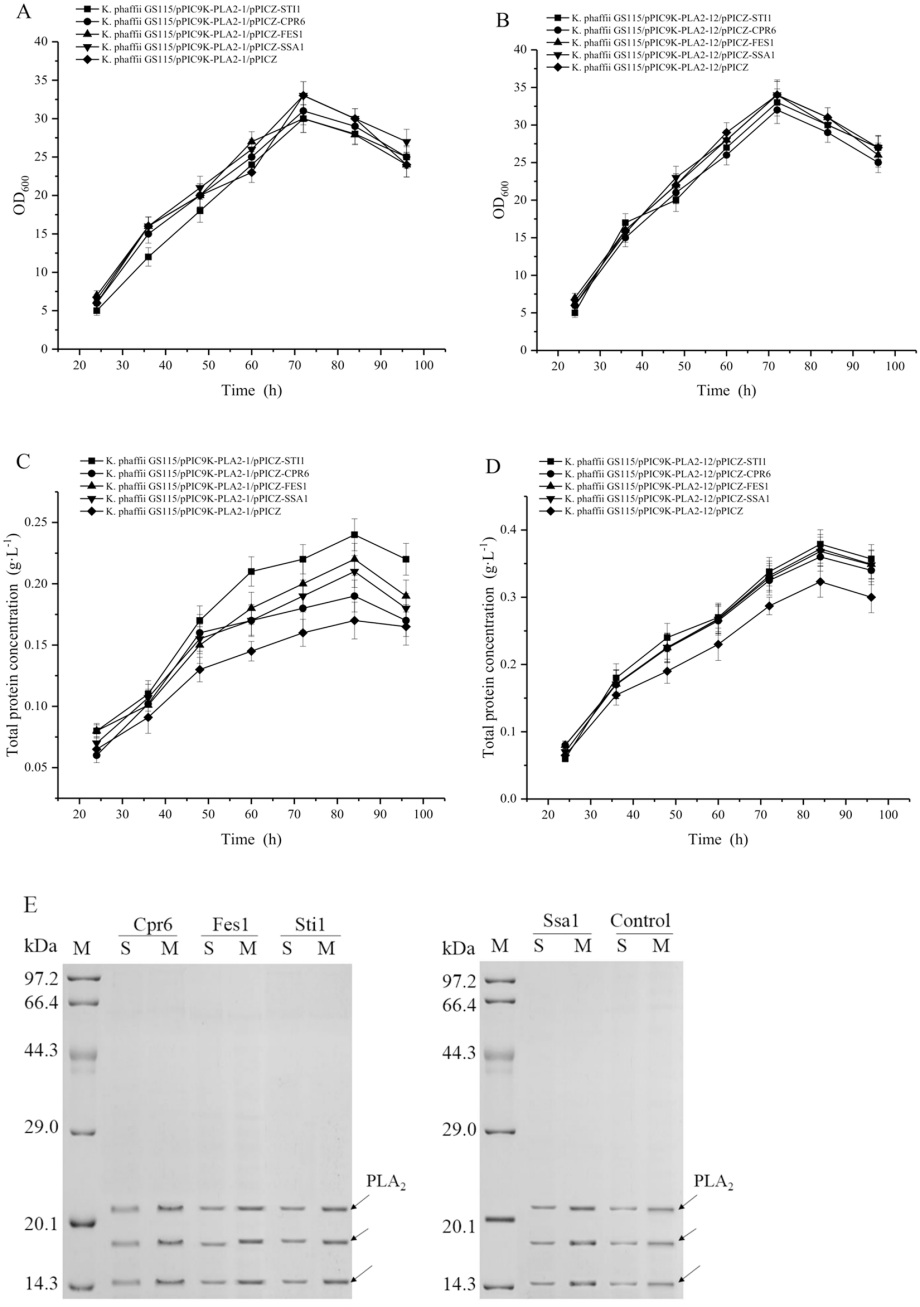
Effect of coexpression of helper genes on PLA_2_ production by *K. phaffii* induced by methanol in shake flasks. The strains harboring 12-copy (*K. phaffii* GS115/pPIC9K-PLA2-12) and 1-copy (*K. phaffii* GS115/pPIC9K-PLA2-1) *PLA*_*2*_ gene were coexpressed with helper genes (*CPR6*, *FES1*, *STI1*, and *SSA1*), respectively. Coexpression of Ssa1 was used as positive control. *K. phaffii* GS115/pPIC9K-PLA2-12/pPICZ and *K*. *phaffii* GS115/pPIC9K-PLA2-1/pPICZ were used as negative control, respectively. Error bars indicate the standard deviation of three biological replicates. (**A,B**) Cell growth profiles; (**C,D**) Total protein concentration in the supernatant; (**E**) SDS-PAGE of total protein in the supernatant; S, M and “Control” indicate the 1-copy strain, the 12-copy strain and the negative control, respectively.

**Figure 7:**
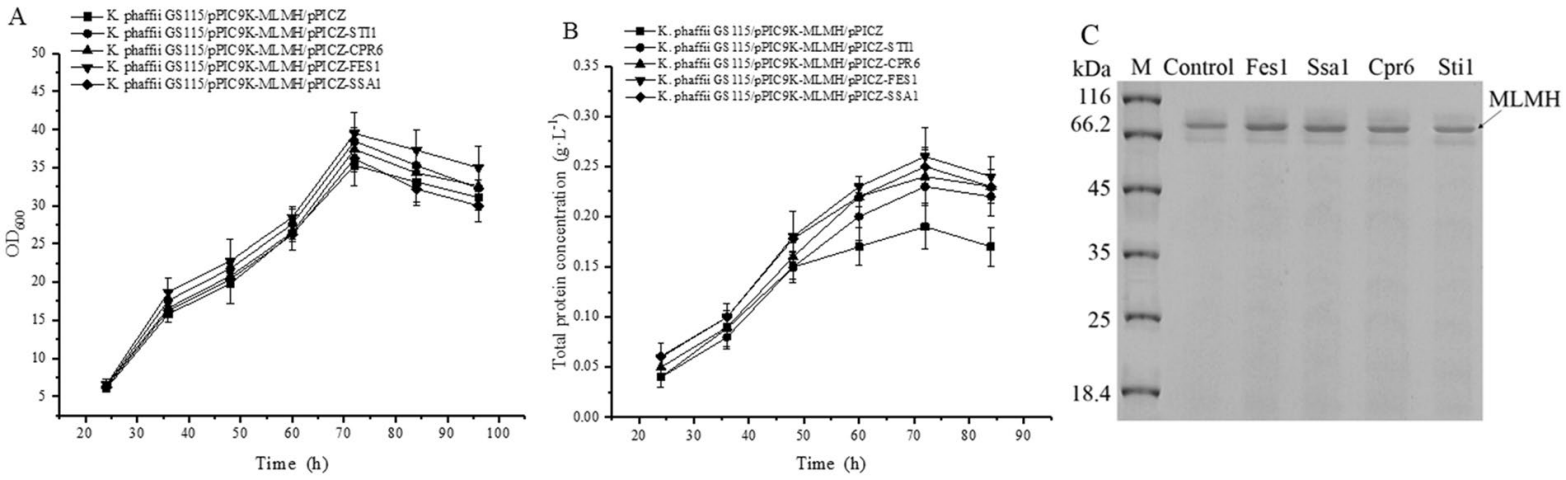
Effect of coexpression of helper genes on MLMH production by *K. phaffii* induced by methanol in shake flasks. (**A**) Cell growth profiles; (**B**) Total protein concentration in the supernatant; (**C**) SDS-PAGE of total protein in the supernatant, and “control” indicates the negative control. *K. phaffii* GS115/pPIC9K-MLMH expressing MLMH was coexpressed with helper genes (*CPR6*, *FES1*, *STI1*, and *SSA1*). Coexpression of Ssa1 was used as positive control. *K. phaffii* GS115/pPIC9K-MLMH/pPICZ were used as negative control. Error bars indicate the standard deviation of three biological replicates.

The potential helper genes (*FES1*, *CPR6*, and *STI1*) among the most significantly upregulated molecular chaperones in response to cellular stress were selected for testing recombinant protein expression changes. Since the same helper gene can have varied effects on different foreign proteins, two reporter enzymes were used; the small size protein of PLA_2_ (14.3KDa) and the larger size protein of MLMH (66 KDa). A single copy of the helper genes were integrated in the genome of the strains of GS115/pPIC9K-PLA2-12, GS115/pPIC9K-PLA2-1 and GS115/pPIC9K-MLMH. The helper genes were expressed under the control of methanol inducible promoter *AOX1 in vivo* and to assess their functions on the production and secretion of reporter enzymes. Coexpression of Ssa1 had a positive effect on the secretory expression of *Candida antarctica* lipase B (CalB)^59^, thus Ssa1 was selected as positive control to compare with the new potential helper factors. The strains expressing PLA_2_ and MLMH integrated with empty vector pPICZ were used as negative control. As for production of PLA_2_, both strains with one and twelve copies of *PLA*_2_ gene were investigated. The results demonstrated that coexpression of helper genes improved PLA_2_ production without influencing the cell growth (Fig. 6A,B). The secreted total protein concentrations in MC were higher than these in SC (Fig. 6C,D). Coexpression of Sti1 showed the highest improvement of PLA_2_ production in the SC strain after 84 h of induction (1.41-fold), followed by Fes1, Ssa1, and Cpr6, but with no significant difference (*p* < 0.05) between the improved value by these helper factors. On the SDS-PAGE loaded with culture supernatant, the amount of PLA_2_ with coexpression of helper factors was also higher compared with the negative control estimated with the Quantity One software (Fig. 6E). The effect of helper genes on the larger size protein of MLMH was investigated. As shown in Fig. 7A, the cell growth rates of all recombinant strains were comparable during the cultivation period. coexpression of Fes1, Ssa1, Cpr6, and Sti1, the total protein concentrations in the supernatant were increased approximately 1.36-, 1.31-, 1.26- and 1.21-fold compared to the control (Fig. 7B), but with no significant difference (*p* < 0.05) between the improved value by these helper factors. The increased expression level is in agreement with the higher amount of MLMH with coexpression on SDS-PAGE (Fig. 7C). When coexpressed with helper genes, the transcription levels of reporter enzymes and helper genes were detected by RT-qPCR (Fig. S4), which indicated that all of the transcription levels of reporter enzymes (Fig. S4A for PLA_2_ and Fig. S4C for MLMH) were higher than that of the control. The reason might be that the cell stress was relieved by coexpression with helper factors, and the better physiology status promoted the transcription level of recombinant proteins. Meanwhile, the transcription levels of helper genes (Fig. S4B and Fig. S4D) all increased upon coexpression, while no obvious trends could be observed.

The selected helper factors in this study are all chaperones or co-chaperones among the Hsp70 and Hsp90 chaperone families, which play key role in protein folding and degradation during heat shock response^50^. Cytoplasmic Hsp70 of Ssa family, especially Ssa1, are involved in the degradation of a variety of misfolded proteins in yeast^60^. The nucleotide exchange factor Fes1, triggers the release of the misfolded proteins from Hsp70, is essential for ubiquitin-dependent degradation of misfolded cytosolic proteins^61^. Overproduction of recombinant proteins is prone to activate the unfolded protein response (UPR), as the cellular machinery is not necessarily prepared to deal with the folding and secretion of overloaded foreign proteins^4^. In our study, the known UPR targets (*KAR*2*, LHS1, JEM1, RFT1*) were significantly upregulated in the 12-copy strain, indicating the activation of UPR. UPR restores ER homeostasis by degrading misfolded proteins, inhibiting translation, and increasing expression of chaperones that enhance ER protein folding capacity^62^. As molecular chaperones, both Ssa1 and Fes1 function in degradation of misfolded protein. Thus, coexpression of Ssa1 or Fes1 would help accelerate the clearance of misfolded proteins and maintain the proper function of ER, and in turn contribute to the production of the reporter enzymes (PLA_2_ and MLMH). It has been reported that when coexpression of Ssa1 the expression of CalB^59^ or a hormone-like protein (G-CSF)^63^ in *K. phaffii* was increased by 1.4-fold and 6.8-fold, respectively. Kar2, one of the key Hsp70s presented in cytosolic and ER luminal, was chosen for many co-overexpression experiments^11^. The overexpression of Kar2 enhanced the production of G-CSF in *K. phaffii* by 5.6-fold^63^, while it showed a negative effect on the expression of CalB^59^.

ATP-dependent Hsp90 molecular chaperone family is a global cellular regulator critical for the folding and regulation of a wide array of cellular proteins^64–66^. Hsp90 functions downstream of HSP70 in the structural maturation and conformational regulation of substrate proteins^50,65,66^. Several regulators and co-chaperones, such as Sti1, Cpr6, Cyp40/Cpr7, Cdc37, Aha1, p23, participate in this process^66^. Among them, Sti1 provides a direct link between Hsp70 and Hsp90, allowing substrate transfer. Cpr6 and Aha1 synergistically stimulate Hsp90’s ATPase activity. These factors are thought to adjust the kinetic properties of the cycle to achieve certain conformational transitions in HSP90-bound substrates, as well as their release from HSP90^50,65,66^. Through binding unstable proteins Hsp90 buffers proteotoxic stress and prevents protein inactivation or aggregation^64–66^. In our study, the Hsp90 co-chaperones, Sti1and Cpr6, were among the top hits of upregulated genes and the transcription level of Hsp90 chaperone (Hsp82) was also improved by 6.3-fold when increasing the copy number of *PLA*_*2*_ from one to twelve in *K. phaffii*. Based on these facts, overexpression of Sti1 or Cpr6 may have positive effect on the dynamic ATP-dependent cycle of Hsp90, which yields global benefits on the cell physiology resulting in the increased secretion of heterologous enzymes. Reports on the effects of coexpression of Hsp90 and its co-chaperones on heterologous protein production are rare. Biette *et al*.^67^ demonstrated that coexpression of the Hsp90 chaperone machinery (Hsp90, Hsp70, Hop/Sti1, Hsp40, and p23) elicited a 2-fold increase in a client protein expression in a baculovirus expression system.

In our study, coexpression of three novel helper genes (*FES1*, *CPR6*, and *STI1*) identified by transcriptomic analysis successfully improved the production of two reporter enzymes in *K. phaffii*. However, the production of two reporter enzymes was only increased to a limited level up to 1.41-fold. Two major reasons might be considered for this relative low improvement. Firstly, molecular chaperones form a complicated interaction network. The systematic analysis of all known 63 chaperones in *S. cerevisiae* demonstrated that molecular chaperones formed multi-component chaperone modules, which could either act on single or multiple folding pathways^68^. Hsp70, Hsp40, Hsp90 and Hsp90 cochaperones cooperate synergistically assist the regulation and folding of client proteins^68,69^. Thus, the effect of coexpression of a single molecular chaperon on the heterologous protein production is limited, which could be solved in a certain degree by coexpression of several closely correlated molecular chaperones. In consideration of interaction between Ydj1 and Ssa1, coexpression of Ydj1/Ssa1 pair increased the production of CalB in *K. phaffii* by 2.5-fold, while individual coexpression of Ydj1 or Ssa1 only increased the CalB production by 1.6- or 1.4-fold, respectively^59^. Zhang *et al*.^63^ reported that the combination of Ydj1/Pdi, Ydj1/Sec63, and Kar2/Pdi chaperones synergistically increased the secretion levels of the target protein by 8.7, 7.6, and 6.5 times, respectively, which were higher than that with individual chaperone. Secondly, successful production of heterologous protein depends on the efficiency of each module of transcription, protein synthesis and translocation, protein folding and quality control, vesicular trafficking and ERAD capacity, etc.^18^. Coexpression of a single helper factor only manipulates one module of protein production, while other steps of protein processing may be still the bottleneck for efficient production of recombinant proteins. Gu *et al*.^13^ alleviated bottlenecks of recombinant protein overexpression by combined coexpression of several genes involved in protein folding genes (*ERO1, CNE1* and *KAR2*), syntaxin and vesicular trafficking-related genes (*SSA4, SSO2, SEC 53* and *BMH2*), ERAD genes (*HRD1* and *UBC1*), and general stress response genes (*GCN4*). Thus, according to above mentioned limitations, further research work could focus on how to enhance other key helper proteins synergistically combined with these novel helper factors involved in heat shock response in this study.

## Electronic supplementary material

Supplementary information
