# Supplementary material for: Identification of novel factors enhancing recombinant protein production in multi-copy Komagataella phaffii based on transcriptomic analysis of overexpression effects
Source: Sci Rep. 2017 Nov 24;7:16249. doi: 10.1038/s41598-017-16577-x (PMC5701153; doi:10.1038/s41598-017-16577-x)
Supplement: Supplementary file 1 — Supplementary information [file 41598_2017_16577_MOESM1_ESM.pdf]

Supplementary information

Identification of novel factors enhancing recombinant protein production in multi-copy *Komagataella phaffii* based on transcriptomic analysis of overexpression effects

Xiao-Wei Yu [1*], Wei-Hong Sun[1], Ying-Zheng Wang[1], Yan Xu [1*]

[1]The Key Laboratory of Industrial Biotechnology, Ministry of Education, School of Biotechnology, Jiangnan University, Wuxi 214122, P. R. China

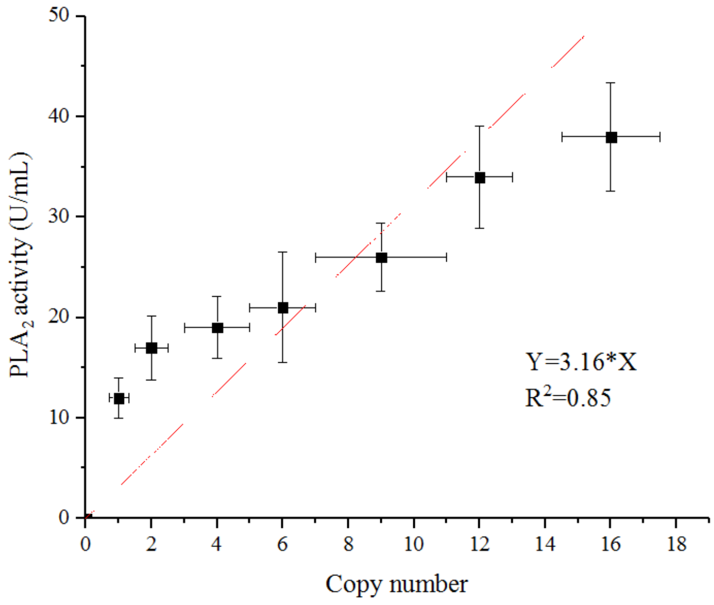

Figure S1. Experimental values for the PLA_2 activity and gene copy number as well as linear regression (red dashed line) for modeling the relationship between the PLA_2 activity and gene copy number. Clones for each multi-copy strain were cultured in shake flask, and the PLA_2 activity was measured at 84 h after methanol induction. Error bars indicate the standard deviation of six biological replicates.

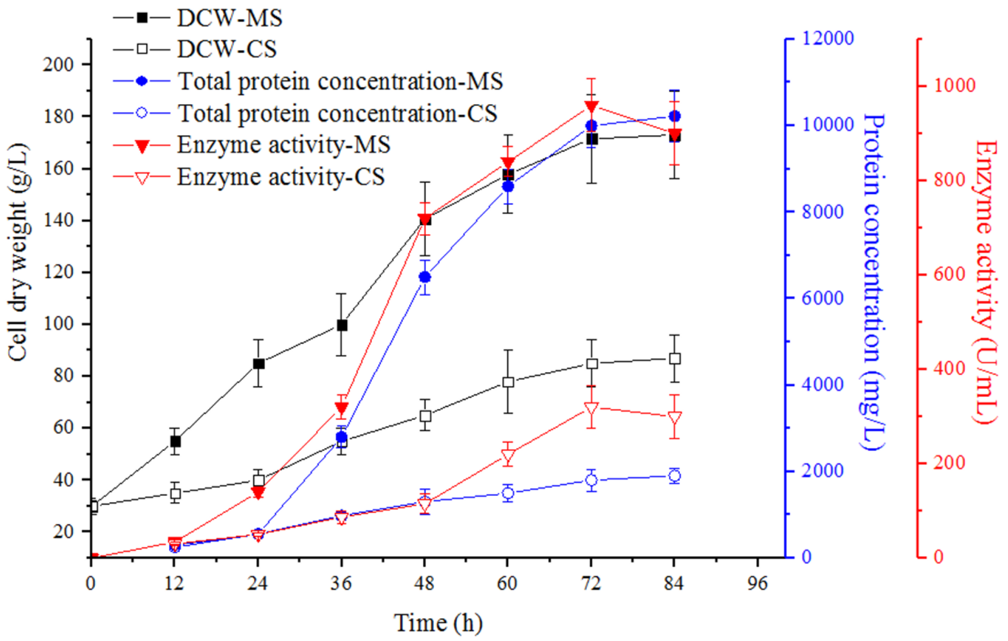

Figure S2. Cell concentration, PLA_2 activity, total protein concentration profiles of GS115/pPIC9K-PLA2-12 (MC) and GS115/pPIC9K-PLA2-1 (SC) during the methanol induction phase. GS115/pPIC9K-PLA2-12 harboring 12-copy PLA_2 gene and GS115/pPIC9K-PLA2-1 harboring 1-copy PLA_2 gene were cultured in fed-batch fermentation. Error bars indicate the standard deviation of three biological replicates.

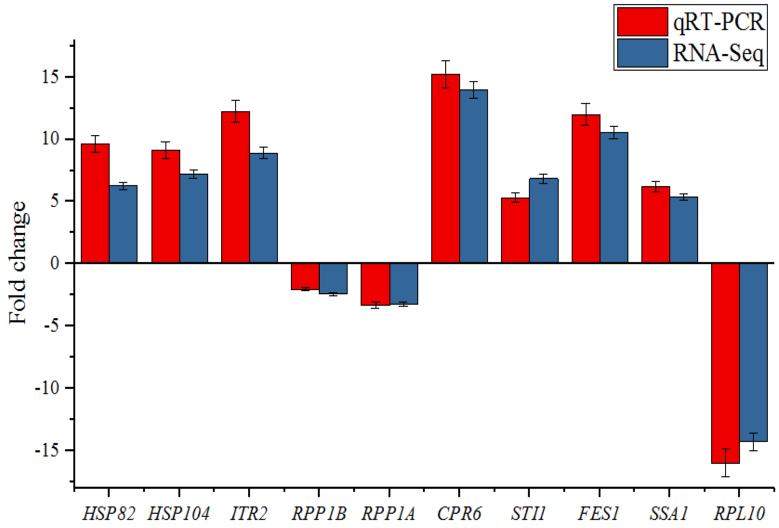

Figure S3. Validation of RNA-seq data by RT-qPCR. RNA-seq and RT-qPCR analyses were performed as described in the experimental section. Relative (RT-qPCR and FPKM (RNA-seq)) expression values are compared between MC and SC and presented as fold change with an average ± SD (standard deviation) of three biological replicates for qRT-PCR data and two biological replicates for RNA-seq data.

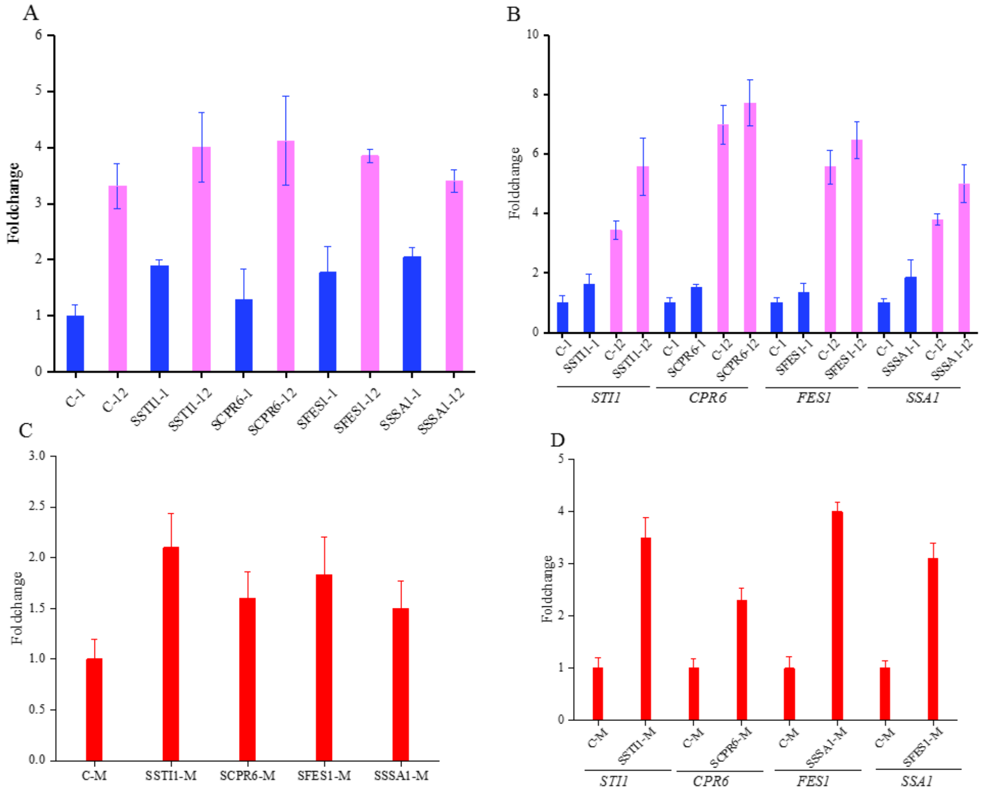

Figure S4. Relative transcription levels of report enzyme genes and coexpressed helper genes in the recombinant strains determined by RT-qPCR. The recombinant strains from which the transcription levels of the indicated genes were determined are labelled on the x-axis. The strain names are indicated in Table S3. The strains with single and twelve-copy of *PLA*$_2$ gene are marked in blue and purple, respectively. The strains expressing MLMH are marked in red. The transcription levels of the genes in the control strains (C-1 and C-M) are set as 1. A: The relative transcription level of *PLA*$_2$ gene in the recombinant strains coexpressing helper factors; B: The relative transcription level of the helper genes in the recombinant strains expressing PLA$_2$ and the helper genes were indicated at the bottom; C: The relative transcription level of *MLMH* gene in the recombinant strains coexpressing helper factors; D: The relative transcription level of the helper genes in the

recombinant strains expressing MLMH and the helper genes were indicated at the bottom. Error bars indicate the standard deviation of three biological replicates.

Table S1

List of top significantly differentially expressed genes in MC vs SC involved in protein processing in ER and heat shock response

Category	Gene name of the corresponding sequence in *S. cerevisiae*/ Gene ID in *K. phaffii* GS115	Gene description	Fold change (MC vs SC)
Protein processing in ER	*HSP82* (PAS_chr1-4_0130)	Hsp90 chaperone, ATP-dependent molecular chaperone，Expressed constitutively and induced by high temperatures	6.26
	KAR2 (PAS_chr2-1_0140)	Hsp70 chaperone, involved in protein import into the ER, mediate protein folding, regulates the unfolded protein response	5.90
	CNE1 (PAS_chr2-1_0322)	Calnexin, integral membrane ER chaperone involved in folding and quality control of glycoproteins	5.53
	SSA1 (PAS_chr4_0552)	Hsp70 chaperone, ATPase involved in protein folding and NLS-directed nuclear transport	5.39
Heat shock response	*CPR6* (PAS_chr3_0567)	Peptidyl-prolyl cis-trans isomerase (cyclophilin); binds to Hsp82p and contributes to chaperone activity	13.98
	FES1 (PAS_chr2-1_0042)	Hsp70 (Ssa1p) nucleotide exchange factor; cytosolic homolog of Sil1p, which is the nucleotide exchange factor for BiP (Kar2p) in the endoplasmic reticulum	10.56
	HSP104 (PAS_chr1-3_0102)	Cooperates with Hsp40 and Hsp70 to refold and reactivate previously denatured, aggregated proteins, responsive to heat stress and DNA replication stress	7.22
	STI1 (PAS_chr2-1_0518)	Hsp90 cochaperone, interacts with the Ssa group of the cytosolic Hsp70 chaperones and activates Ssa1p ATPase activity	6.84

Table S2

List of gene information of the differentially expressed genes in MC vs SC involved in ribosome, DNA replication, protein processing and heat shock response in Figure 5

Gene ID in *K. phaffii* GS115	Gene name of the corresponding sequence in *S. cerevisiae*	Gene description
Category: Ribosome		
PAS_chr1-4_0358	*MNP1*	Protein associated with the mitochondrial nucleoid
PAS_chr3_0946	*RSM10*	Mitochondrial ribosomal protein of the small subunit, has similarity to E. coli S10 ribosomal protein; essential for viability
PAS_chr4_0845	*MRPL32*	Mitochondrial ribosomal protein of the large subunit; protein abundance increases in response to DNA replication stress
PAS_chr3_0335	*RPL43B*	Ribosomal 60S subunit protein L43B; protein abundance increases in response to DNA replication stress
PAS_chr4_0412	*RPP1A*	Ribosomal stalk protein P1 alpha, involved in the interaction between translational elongation factors and the ribosome
PAS_chr4_0982	*RPP1B*	Ribosomal protein P1 beta, component of the ribosomal stalk, which is involved in interaction of translational elongation factors with ribosome
PAS_chr2-1_0634	*RPS27B*	Protein component of the small (40S) ribosomal subunit
PAS_chr4_0180	*RPL31B*	Ribosomal 60S subunit protein L31B; associates with karyopherin Sxm1p
PAS_chr3_0194	*RPL42B*	Ribosomal 60S subunit protein L42B; required for propagation of the killer toxin-encoding M1 double-stranded RNA satellite of the L-A double-stranded RNA virus
PAS_chr1-4_0412	*RPL38*	Ribosomal 60S subunit protein L38
PAS_chr2-2_0054	*RPL10*	Ribosomal 60S subunit protein L10; responsible for joining the 40S and 60S subunits; regulates translation initiation
Category: DNA replication		

PAS_chr4_0223	*MCM2*	Protein involved in DNA replication; component of the Mcm2-7 hexameric complex that binds chromatin as a part of the pre-replicative complex; relative distribution to the nucleus increases upon DNA replication stress
PAS_chr2-1_0149	*MCM3*	Protein involved in DNA replication; component of the Mcm2-7 hexameric complex that binds chromatin as a part of the pre-replicative complex
PAS_chr3_0233	*POL30*	Proliferating cell nuclear antigen (PCNA), functions as the sliding clamp for DNA polymerase delta; may function as a docking site for other proteins required for mitotic and meiotic chromosomal DNA replication and for DNA repair
PAS_chr1-3_0083	*POL2*	Catalytic subunit of DNA polymerase (II) epsilon, a chromosomal DNA replication polymerase that exhibits processivity and proofreading exonuclease activity; also involved in DNA synthesis during DNA repair; interacts extensively with Mrc1p
PAS_chr4_0323	*PRI1*	Subunit of DNA primase, which is required for DNA synthesis and double-strand break repair
PAS_chr3_0765	*DPB2*	Second largest subunit of DNA polymerase II (DNA polymerase epsilon), required for maintenance of fidelity of chromosomal replication; expression peaks at the G1/S phase boundary; Cdc28p substrate
Category: Protein processing in ER		
PAS_chr1-4_0130	*HSP82*	Hsp90 chaperone; redundant in function with Hsc82p; required for pheromone signaling, negative regulation of Hsf1p
PAS_chr2-1_0322	*CNE1*	Calnexin, integral membrane ER chaperone involved in folding and quality control of glycoproteins
PAS_chr2-2_0015	*JEM1*	DnaJ-like chaperone required for nuclear membrane fusion during mating, localizes to the ER membrane; exhibits genetic interactions with KAR2
PAS_chr2-2_0066	*YDJ1*	Type I HSP40 co-chaperone; involved in regulation of HSP90 and HSP70 functions
PAS_chr4_0552	*SSA1*	Hsp70 chaperone, ATPase involved in protein folding and NLS-directed nuclear transport
PAS_chr2-1_0140	*KAR2*	Hsp70 chaperone, involved in protein import into the ER, mediate protein folding, regulates the unfolded protein response

PAS_chr1-3_0063	*LHS1*	Molecular chaperone of the endoplasmic reticulum lumen, involved in polypeptide translocation and folding; nucleotide exchange factor for the ER lumenal Hsp70 chaperone Kar2p; regulated by the unfolded protein response pathway
Category: Heat shock protein		
PAS_chr2-1_0518	*STI1*	Hsp90 cochaperone, interacts with the Ssa group of the cytosolic Hsp70 chaperones and activates Ssa1p ATPase activity
PAS_chr1-3_0102	*HSP104*	Cooperates with Hsp40 and Hsp70 to refold and reactivate previously denatured, aggregated proteins, responsive to heat stress and DNA replication stress
PAS_chr2-1_0324	*HSP78*	Oligomeric mitochondrial matrix chaperone that cooperates with Ssc1p in mitochondrial thermotolerance after heat shock; able to prevent the aggregation of misfolded proteins as well as resolubilize protein aggregates
PAS_chr3_0170	*HCH1*	Heat shock protein regulator; binds to Hsp90p and may stimulate ATPase activity
PAS_chr1-4_0130	*HSP82*	Hsp90 chaperone, ATP-dependent molecular chaperone，Expressed constitutively and induced by high temperatures
PAS_chr2-1_0828	*CDC37*	Essential Hsp90p co-chaperone; necessary for passage through the START phase of the cell cycle; stabilizes protein kinase nascent chains and participates along with Hsp90p in their folding
PAS_chr1-4_0072	*AHA1*	Co-chaperone that binds to Hsp82p and activates its ATPase activity; similar to Hch1p; expression is regulated by stresses such as heat shock
PAS_chr2-2_0066	*YDJ1*	Type I HSP40 co-chaperone; involved in regulation of HSP90 and HSP70 functions; critical for determining cell size at Start as a function of growth rate
PAS_chr1-4_0043	*SBA1*	Co-chaperone that binds to and regulates Hsp90 family chaperones; important for pp60v-src activity in yeast
PAS_chr2-2_0323	*MDJ1*	Co-chaperone that stimulates the ATPase activity of the HSP70 protein Ssc1p; involved in protein folding/refolding in the mitochodrial matrix; required for proteolysis of misfolded proteins; member of the HSP40 (DnaJ) family of chaperones

PAS_chr4_0552	*SSA1*	ATPase involved in protein folding and NLS-directed nuclear transport; member of HSP70 family; forms chaperone complex with Ydj1p
PAS_chr3_0567	*CPR6*	Peptidyl-prolyl cis-trans isomerase (cyclophilin); binds to Hsp82p and contributes to chaperone activity
PAS_chr2-1_0042	*FES1*	Hsp70 (Ssa1p) nucleotide exchange factor; cytosolic homolog of Sil1p, which is the nucleotide exchange factor for BiP (Kar2p) in the endoplasmic reticulum
PAS_chr4_0627	*HSP12*	Plasma membrane protein involved in maintaining membrane organization in stress conditions; induced by heat shock, oxidative stress, osmostress, stationary phase, glucose depletion, oleate and alcohol

Table S3

List of *K. phaffii* recombinant strains coexpressed with helper genes

Strain name	Strain and integrated plasmid	Recombinant protein / Gene copy number	Coexpressed with helper genes
C-1	*K. phaffii* GS115/pPIC9K-PLA-1/pPICZ	PLA_2/1	Without coexpression
C-12	*K. phaffii* GS115/pPIC9K-PLA-12/pPICZ	PLA_2/12	Without coexpression
SSTI1-1	*K. phaffii* GS115/pPIC9K-PLA-1/pPICZ-STI1	PLA_2/1	Coexpressed with *STI1*
SSTI1-12	*K. phaffii* GS115/pPIC9K-PLA-12/pPICZ-STI1	PLA_2/12	Coexpressed with *STI1*
SCPR6-1	*K. phaffii* GS115/pPIC9K-PLA-1/pPICZ-CPR6	PLA_2/1	Coexpressed with *CPR6*
SCPR6-12	*K. phaffii* GS115/pPIC9K-PLA-12/pPICZ-CPR6	PLA_2/12	Coexpressed with *CPR6*
SFES1-1	*K. phaffii* GS115/pPIC9K-PLA-1/pPICZ-FES1	PLA_2/1	Coexpressed with *FES1*
SFES1-12	*K. phaffii* GS115/pPIC9K-PLA-12/pPICZ-FES1	PLA_2/12	Coexpressed with *FES1*
SSSA1-1	*K. phaffii* GS115/pPIC9K-PLA-1/pPICZ-SSA1	PLA_2/1	Coexpressed with *SSA1*
SSSA1-12	*K. phaffii* GS115/pPIC9K-PLA-12/pPICZ-SSA1	PLA_2/12	Coexpressed with *SSA1*
C-M	*K. phaffii* GS115/pPIC9K-MLMH/pPICZ	MLMH/1	Without coexpression
SSTI1-M	*K. phaffii* GS115/pPIC9K-MLMH/pPICZ-STI1	MLMH/1	Coexpressed with *STI1*
SCPR6-M	*K. phaffii* GS115/pPIC9K-MLMH/pPICZ-CPR6	MLMH/1	Coexpressed with *CPR6*
SFES1-M	*K. phaffii* GS115/pPIC9K-MLMH/pPICZ-FES1	MLMH/1	Coexpressed with *FES1*
SSSA1-M	*K. phaffii* GS115/pPIC9K-MLMH/pPICZ-SSA1	MLMH/1	Coexpressed with *SSA1*
